# Correlation of Vascularization and Inflammation with Severity of Oral Leukoplakia

**Published:** 2017-04-01

**Authors:** Fatemeh Mashhadiabbas, Masoume Fayazi-Boroujeni

**Affiliations:** 1 *Dept. of Oral and Maxillofacial Pathology, Dental school, Shahid Beheshti University of Medical Sciences, Tehran, Iran*; 2 *Shahrekord University of Medical Sciences, Shahrekord, Iran*

## Abstract

**Background & objective:**

Changes in submucosal vascularization and inflammation, determined by immunohistochemistry staining, were shown to be correlated with the development of dysplasia and invasiveness of epithelial cells in premalignant and malignant lesions. This study evaluated changes in sections routinely stained with Hematoxylin and Eosin (H&E) in order to investigate vascular density and intensity of inflammatory cells infiltration during the progression of oral leukoplakia with mild dysplasia to Squamous Cell Carcinoma (SCC).The aim of the research was to determine whether changes in sub-mucosal vascularity and inflammatory infiltration of leukoplakia in routine H&E-stained sections could contribute to the assessment of severity of the lesion.

**Methods:**

In this cross-sectional, comparative and descriptive study, vascular density and inflammation intensity of 125 available samples of H&E-stained sections, consisting of 35 cases of mild and moderate dysplasia, 38 severe dysplasia and carcinoma in situ, and 52 SCC, were investigated. To analyze the data, chi-square test, Mann-Whitney test, Kruskal-Wallis test, Tukey’s post hoc test, and cumulative ordinal logistic regression were conducted.

**Results:**

There was a significantly higher vascular density in cases with severe dysplasia, in situ carcinoma, and SCC compared to those with mild to moderate dysplasia (*P*<0.0001). However, the difference in vascularity was not statically significant between severe dysplasia, carcinoma in situ, and SCC (*P*=0.78). Intensity of inflammatory cells infiltration in the underlying connective tissue was significantly different among the three groups (*P*<0.0001), and the highest intensity of inflammatory cells infiltration was seen in the SCC group.

**Conclusions:**

Increased submucosal vascularization and inflammatory cells infiltration can contribute further to predicting more aggressive epithelial dysplasia.

## Introduction

Leukoplakia is clinically found as a white plaque in the oral mucosa, which is not recognized as any other known lesion. Its histological changes vary from hyperkeratosis, dysplasia, and carcinoma in situ to invasive Squamous Cell Carcinoma (SCC). Approximately 15% of epithelial dysplasia progress to SCC (‎^1^, ‎^2^). It is a frequent oral lesion that is referred to pathologists for diagnosis and grading. The grading system is based on dysplastic changes in epithelial cell layer, yet, changes in the submucosal vascularization (3-10) as well as the inflammation (11-15) have been demonstrated to be correlated with development of dysplasia and invasiveness of epithelial cells in premalignant and malignant lesions. Different studies (5,6,8,10) have shown that increased vascularization occurs at early stages of dysplastic transformation, and there is no significant difference among severe dysplasia, carcinoma in situ, and SCC. All of these studies have used immunohistochemistry staining to examine the intensity of the mentioned submucosal changes, yet, these changes can also be examined in sections stained with hematoxylin and eosin (H&E), as performed by the studies of Jalayer et al. and Gommes et al.(18, 19).

This study investigated whether changes in submucosal vascularity and inflammatory infiltration of leukoplakia in routine H&E-stained sections can contribute to the assessment of severity of the lesion.

## Materials and Methods

The study group included 134 available samples from the Department of Oral and Maxillofacial Pathology, Shahid Beheshti University of Medical Sciences. The samples were histopathologically diagnosed as oral dysplasia or squamous cell carcinoma. None of the patients had received any treatments. The data on age and gender were collected from the patients’ medical files.

Primarily, the H&E-stained sections of all cases were observed using an optical microscope (Olympus,CH-2,Japan) by two experienced oral pathologists and graded according to the World Health Organization (WHO) classification system as mild, moderate, severe dysplasia, carcinoma in situ, early invasive, and SCC (16). The cases with insufficient underlying connective tissue for assessment and with suspected diagnoses such as lichenoid dysplasia and atypia secondary to inflammation were excluded. Finally, 125 samples were selected for examination.

Vascularity was then assessed in inflammation-free areas of the underlying connective tissue adjacent to the epithelial lesion (3) and the invasive edge of the SCC. The sections were scanned at 40X microscopic field (4X objective lens and 10X ocular lens) to determine the areas of higher blood vessel density (hot spots). Next, the mean number of blood vessels with ≤0.5μm of diameter in the five hot spot fields was determined under 400X microscopic field (‎^17^). Moreover, the intensity of inflammation was evaluated and graded as mild (less than 25 inflammatory cells), moderate (25 to 125 inflammatory cells), and severe (greater than 125 inflammatory cells) (‎^17^). 

For the analysis of the data, chi-square test, Mann-Whitney test, Kruskal-Wallis test, Tukey’s posthoc test, and cumulative ordinal logistic regression were performed. Furthermore, cumulative ordinal logistic regression was used for modeling the effect of age, vascular density, and severity of inflammation on the grade of the lesion.

## Results

**Patients and tissue characteristics**

A total of 125 samples, available from the archive, were examined, consisting of three groups: i) mild and moderate dysplasia (35 cases), ii) severe dysplasia and carcinoma in situ (38 cases), and iii) squamous cell carcinoma (52 cases) with patients’ mean ± SD age for each group being 53.4±11.7, 59.93±13.01,and 63.56±14.20 years, respectively. The mean age of the patients was significantly different between the three groups (*P*=0.005); post hoc comparisons revealed a significant difference in age between the mild and moderate dysplasia group and the SCC group (*P*=0.003). 

Moreover, 42.9% (n=15) of patients with mild and moderate dysplasia, 71.4% (*n*=27) of patients with severe dysplasia and carcinoma in situ, and 50% (*n*=26) of patients with SCC were female. No significant association was observed between gender and the grade of the lesion (*P*=0.22).

**Numerical vascular density**

The number of vessels was counted for each tissue. For mild and moderate dysplasia, the mean number ±SD of vessels was 9.46±5.53, for severe dysplasia and carcinoma in situ this was 17.86±6.36, and for SCC a mean number of 19.11±6.50 was recorded. Tukey’s post hoc comparison of the number of vessels in different groups demonstrated a significantly higher vascular density in severe dysplasia, carcinoma in situ, and SCC compared to mild and moderate dysplasia (*P*<0.0001), yet, the vascularity of severe dysplasia and carcinoma in situ was not statistically different from that of SCC (*P*=0.78) (Table 1).

**Inflammatory cells infiltration**

The intensity of inflammatory cells infiltration in the underlying connective tissue of the studied groups was significantly different (*P*<0.0001) (Table 2)

**Table 1:** Mean Vascularity of the Studied Groups

Groups		Mean (SD)	*P *value
Mild to moderate dysplasia		9.46±5.53	
Severe dysplasia to carcinoma		17.86±6.36	0.000^*^
Squamous cell carcinoma		19.12±6.50	

* The vascularity of mild to moderate dysplasia group was significantly lower than the other groups (*P*<0.001)

**Table 2:** Intensity of Inflammatory Cells Infiltration in the Studied Groups

**Inflammation**		**Groups**			**P Value**
		Mild to moderate dysplasia	Severe dysplasia to carcinoma	Squamous cell carcinoma	
**Mild**		18 (51.4%)	0 (0%)	5 (9.6%)	
**Moderate**		9 (25.7%)	11 (28.6%)	9 (17.3%)	0.000^*^
**Severe**		8 (22.9%)	27 (71.4%)	38 (73.1%)	

* There was a significant difference among the three groups (*P*<0.0001).

For modeling the effect of age, vascular density, and severity of inflammation on the grade of lesion, an ordinal logistic regression was performed. The results showed that a lesion with severe inflammation increased the odds of more severe lesions by 4.42 times compared to a lesion with mild inflammation (*P*=0.03). Moreover, the risk of severe lesions increases with age by 1.05 times each year (*P*=0.007), while increase in vascular density is positively correlated with more severe dysplasia *(P*<0.001) (Table 3).

**Table 3:** Ordinal Logistic Regression for Modeling the Effect of Age, Vascular Density, and Severity of Inflammation on the Grade of the Lesion

Variables		B	SE	p-value	OR
Intercept	**grade (** **mild to moderate dysplasia** **)**	4.99	1.25	<0.001	146.78
	**grade (** **severe dysplasia to carcinoma** **)**	5.92	1.29	<0.001	373.62
Inflammation (severe)		1.49	0.69	0.03*	4.42
Inflammation(Moderate)		0.49	0.73	0.55	1.55
Inflammation(Mild)		0	-	-	-
Age		0.05	0.02	0.007*	1.05
Vascularity		0.14	0.04	<0.001*	1.15

* The lesions with severe inflammation increased the odds of more severe lesion compared to lesions with mild inflammation (P=0.03). The risk of more severe lesions increases with age (P=0.007), while, increase in vascular density is positively related to severer dysplasia (P<0.001).

**The Relationship between vascularity and inflammation**

An increase in the number of vessels was seen following elevated intensity of inflammatory cells infiltration in the three groups. Kruskal-Wallis test indicated that the mean number of vessels in lesions with mild intensity of inflammatory cells infiltration was significantly lower than in those with moderate and severe intensity of inflammatory cells infiltration (P<0.05); yet, no significant difference was observed in the mean number of vessels between lesions with moderate and those with severe intensity of inflammatory cells infiltration (Table 4). 

**Table 4:** Comparison of Mean Vascularity at Different Inflammation Intensities

Group	Inflammation	Mean Number of Vessels ±SD	P-Value
Mild to moderate dysplasia	Mild	**7.11±3.60 ** ^(^^1^^)^	**0.024 ** ^*^ (Kruskal- Wallis Test)
	Moderate	**11.89±5.67 ** ^(^^1^^)^	
	Severe	**12.00±7.17 ** ^(^^1^^)^	
Severe dysplasia to carcinoma	Mild	**-----**	**0.454** (Mann-Whitney Test)
	Moderate	**16.00±6.63**	
	Severe	**18.60±6.45**	
Squamous cell carcinoma	Mild	**12.40±3.78 ** ^(^^1^^)^	**0.034 ** ^*^ (Kruskal Wallis Test)
	Moderate	**14.11±5.58 ** ^(^^1^^)^	
	Severe	**20.00±6.56 ** ^(^^1^^)^	

* *P*<0.05

1 The mean vascularization of all groups with mild inflammation was significantly lower than those with moderate and severe inflammation.

## Discussion

The findings of this study on vascular density in H&E stained sections are in agreement with several previous studies (3-10). Although a smaller number of vessels were observed in H&E stained sections compared with immunohistochemically stained ones, yet, a significant difference in vascularization among the three groups was evident. The results indicated a significantly higher vascular density in severe dysplasia, carcinoma in situ, and SCC compared with mild and moderate dysplasia, however, the difference in vascularity between severe dysplasia, carcinoma in situ, and SCC was not statistically significant, yet was higher in SCC. These findings are in agreement with the study of Michailidou *et al.*(5), which reported that the number of micro vessels (stained withCD34) increased significantly in SCC compared to leukoplakia with mild and moderate dysplasia. However, no significant increase in the number of micro vessels was observed between leukoplakia with severe dysplasia and SCC. The results are also in accordance with the study of Gandolfo *et al.*(‎^4^), in which CD34 was used to stain blood vessels and sub-basal vascularization was found to be higher in leukoplakia with dysplasia than in normal mucosa, and the highest density of vascularization was seen in SCC. Hence, they suggested that if the magnitude of the vascularization is sufficient, it can be assessed in H&E-stained sections to evaluate the severity of the lesion. Moreover, based the findings of the current study and in accordance with previous studies (4, 5, 7, 9), it can also be inferred that angiogenesis is mainly induced at the early stages of malignant transformation.

A correlation has long been speculated between inflammation and cancer. It has become apparent that the interaction between transformed cells and the surrounding environment, such as immune cells, can cause promotion and progression of many epithelial tumors (11, 14, 19).

In the present study, it was observed that the intensity of inflammatory cells infiltration increased with elevation in the severity of epithelial dysplasia from mild dysplasia to carcinoma in H&E-stained sections. This finding is consistent with the study of Gannot *et al. *(‎^13^) in which the total number of immune cells infiltration (CD4, CD8 and B cells) was found to be significantly elevated in cases with moderate and severe dysplasia compared with the hyperkeratosis and mild dysplasia. In addition, the mentioned study found that B cells were very prominent in moderate and severe dysplasia and even more prominent in SCC. 

However, Syafriadi *et al.*(15) reported that the number of T cells increases in severe dysplasia and carcinoma in situ, yet it decreases in carcinomas. This inconsistency in the findings can be explained by the fact that this study evaluated all chronic inflammatory cells, including both T and B cells. In contrast to T cells, the number of other chronic inflammatory cells, such as B cells, has been found to be increasing with the severity of lesion, as reported by Gannot *et al.*(‎^13^). Therefore, the total number of chronic inflammatory cells increases with the severity of lesion despite the decrease of T cells.

In Addition to tumor cells that promote angiogenesis, tumor-associated inflammatory cells can also contribute to this process by a variety of growth factors such as Vascular Endothelial Growth Factor (VEGF) (that can augment pro-inflammatory T cell differentiation), cytokines and proteases (20, 21). Accordingly, a correlation was expected between increased inflammatory cells infiltration and mean number of vessels, although this study sought to count vessels in inflammation-free areas. The assessment of the correlation between the intensity of inflammatory cells infiltration and vascular density in the underlying connective tissue of the studied groups revealed that the mean number of vessels in the samples with mild intensity of inflammatory cells infiltration was significantly lower than the other samples. In fact, the current study observed a correlation between vascularity and inflammation in the early stage of dysplastic changes, not in the invasive stage. To the best of our knowledge, no study has yet investigated this correlation.

## Conclusion

The results of this study revealed that the observation of the submucosal vascularity and inflammatory infiltration of leukoplakia in routine H&E-stained sections could be used to assess severity of the lesion. In particular, observation of high vascular density and inflammation in the submucosa of H&E-stained sections of oral leukoplakia predicts more aggressive epithelial dysplasia.
